# Physical Activity and Gastric Cancer Risk in Patients with and without *Helicobacter pylori* Infection in A Korean Population: A Hospital-Based Case-Control Study

**DOI:** 10.3390/cancers10100369

**Published:** 2018-10-02

**Authors:** Madhawa Neranjan Gunathilake, Jeonghee Lee, Aelee Jang, Il Ju Choi, Young-Il Kim, Jeongseon Kim

**Affiliations:** 1 Department of Cancer Control and Population Health, Graduate School of Cancer Science and Policy, Goyang-si 10408, Gyeonggi-do, Korea; 1806104@ncc.re.kr; 2 Department of Cancer Biomedical Science, Graduate School of Cancer Science and Policy, Goyang-si 10408, Gyeonggi-do, Korea; jeonghee@ncc.re.kr (J.L.); jal0008@naver.com (A.J.); 3 Center for Gastric Cancer, National Cancer Center Hospital, National Cancer Center, Goyang-si 10408, Gyeonggi-do, Korea; cij1224@ncc.re.kr (I.J.C.); 11996@ncc.re.kr (Y.-I.K.)

**Keywords:** physical activity, gastric cancer, *Helicobacter pylori* infection

## Abstract

Although physical activity (PA) is beneficial for prolonging lifespan, evidence for the protective role of PA against the development of gastric cancer (GC) is not yet well established. This study assessed the association between PA and GC risk in patients with and without *Helicobacter pylori (H. pylori)* infection in a Korean population. In total, 415 GC patients and 830 controls were enrolled at the National Cancer Center, Korea. The International Physical Activity Questionnaire-Short Form was used to collect PA data. The odds ratios (ORs) and 95% confidence intervals (CIs) were estimated using unconditional logistic regression models. In the *H. pylori*-positive group, subjects who engaged in regular exercise showed a significantly reduced risk of GC in the entire population (OR = 0.52, 95% CI = 0.38–0.70) and in females (OR = 0.60, 95% CI = 0.21–0.64). Subjects who engaged in a high level of total PA showed a significantly reduced risk of GC relative to subjects in the lowest tertile in the fully adjusted model (OR = 0.46, 95% CI = 0.32–0.65, *p*-trend < 0.001). There was an inverse association between PA and GC risk in the entire population, and in the *H. pylori*-positive subgroup. Our data indicate the need for the promotion of all domains of PA, especially for Korean populations.

## 1. Introduction

Gastric cancer (GC) represents the fifth-most commonly diagnosed cancer and the third-leading cause of cancer-related deaths worldwide [1,2], although overall GC incidence rates have been decreasing substantially in most parts of the world for decades [3,4].

Recent global cancer statistics show that the estimated incidence rates of GC are markedly elevated in East Asia with values of 32.1 and 13.2 per 100,000 for men and women, respectively [1]. In Korea, the age-standardized incidence rate of GC was 33.8 per 100,000 across both sexes and 49.3 and 20.5 per 100,000 for men and women, respectively [5]. A study on the prediction of cancer incidence and mortality in Korea identified stomach cancer as the second-most common type of cancer in the country, which represents a significant proportion of the overall burden of cancer among Koreans [6]. Several associated risk factors for GC have been identified including infection with *Helicobacter pylori* (*H. pylori*); male sex; lifestyle factors such as diet, tobacco smoking, and excessive alcohol drinking; family history of GC; and previous stomach surgery [7,8,9,10,11,12,13,14,15]. Of these factors, infection with *H. pylori*, the best-known risk factor for GC, is endemic throughout East Asia. However, only approximately 1% of *H. pylori*-infected subjects develop GC [16,17,18]. In contrast, more than 90% of GC patients have current or past *H. pylori* infection [18]. Therefore, a decrease in chronic *H. pylori* infection is considered to be a main preventive strategy against GC [16]. Interestingly, a reduction in stomach cancer rates has been identified as resulting, in part, from factors related to the increased use and availability of refrigeration, including an increased availability of fresh fruits and vegetables and a reduced consumption of salted and preserved foods [7]. Although there is limited evidence for the influence of other lifestyle factors, it is important to consider lifestyle factors. particularly physical activity (PA). that might be effective in the primary prevention of the disease [19]. A lack of PA, a potentially modifiable lifestyle risk factor, has been associated with increased incidences of cancers of the colon [20,21], breast [22], lung [23], and endometrium [24]. PA can be effective in the primary prevention of some cancers and can reduce the risk of developing some cancers [25]. However, whether PA reduces the risk of other cancers remains unclear. Although many epidemiological studies have reported an association between PA and GC, their findings have been inconsistent, and the degrees of association are modest [19]. It has been recommended that engaging in a minimum of 150 min per week of moderate-intensity PA or 75 min per week of vigorous-intensity PA is beneficial for a healthy life [26]. However, it remains unclear whether the extent of PA influences the prevention of GC [27]. Thus, the utilization of domain-specific PA, considering both its frequency and duration, to address the influences of PA on health needs is needed. Furthermore, it is important to investigate whether and how PA reduces GC risk through its association with innate immunity and potentially affects susceptibility to infections and cancer. A previous study concluded that regular moderate physical activity might lead to lowered susceptibility to viral and bacterial infections [28]. Moreover, exercise-induced protective effects against cancer have been observed in animal models of infection depending on the exercise dosage and the infectious disease model [29,30,31].

Therefore, in this case-control study, we aimed to verify the associations between both the frequency and duration of PA and GC risk by sex and by *H. pylori* infection status.

## 2. Results

Table 1 presents the general characteristics of the study participants with and without GC. The proportion of current smokers was higher in the case subjects (30.8%) than in the controls (19.5%), whereas the proportion of nonsmokers was lower in the case subjects (40.2%) than in the controls (46.3%) (*p* < 0.001). The proportion of nondrinkers was similar between the case subjects (28.7%) and controls (28.4%). The case subjects were more likely to have a family history of GC (*p* < 0.001). Case subjects engaged in less regular exercise (*p* < 0.001), were less educated (*p* < 0.001), and exhibited lower employment rates (*p* < 0.001) and lower monthly incomes (*p* < 0.001) than controls. The proportion of *H. pylori* seropositivity among the case subjects (92.1%) was higher than that among the controls (58.6%). The walking Metabolic Equivalents (MET) minutes per week was lower in the case group than in the control group (*p* = 0.001). The proportions of case subjects with moderate (30.8%) and high (27.0%) levels of PA were lower than those of controls (moderate 37.2%, high 29.9%) (*p* = 0.005). In contrast, the proportion of low PA was higher (42.2%) among case subjects than among controls (32.9%). Case subjects had a higher energy intake than did controls (*p* < 0.001). Compared with men and women in the case group, those in the control group had higher education levels, and monthly incomes, were more likely to have jobs, were less likely to be smokers, engaged in more regular exercise, had higher walking MET minutes per week, and had lower proportions of *H. pylori* infection. A lower proportion of men had a family history of GC in the control group than in the case group, and higher proportions of men engaged in high levels of PA and had lower total energy intake in the control group than in the case group.

Table 2 shows the association between regular exercise and GC risk according to sex. Subjects who engaged in regular exercise showed a significantly reduced risk of GC relative to those who did not engage in regular exercise in model 2 (OR = 0.47, 95% CI = 0.36–0.62), which was adjusted for smoking, first-degree family history of GC, education, occupation, monthly income, and total energy intake. A similarly reduced risk was observed for model 3 (OR = 0.52, 95% CI = 0.39–0.69), which was additionally adjusted for *H. pylori* infection. A significantly lower risk of GC with regular exercise was observed for males in model 2 (OR = 0.65, 95% CI = 0.46–0.91), whereas the decrease in risk with exercise was not significant after further adjusting for *H. pylori* infection. Among females, a significant inverse association between regular exercise and GC risk was observed for model 2 (OR = 0.30, 95% CI = 0.18–0.48) and model 3 (OR = 0.34, 95% CI = 0.21–0.57). Table S1 presents the corresponding associations after stratification by *H. pylori* infection. In the *H. pylori*-positive group, subjects who engaged in regular exercise showed a significantly reduced risk of GC in all study populations (OR = 0.52, 95% CI = 0.38–0.70) and in females (OR = 0.36, 95% CI = 0.21–0.64) after adjusting for possible confounding factors. However, in the *H. pylori*-negative group, a reduced risk of GC with exercise was observed only for females (OR = 0.14, 95% CI = 0.03–0.68).

Table 3 presents the association between PA and GC risk based on the International Physical Activity Questionnaire-Short Form (IPAQ-SF) categorical score. PA was categorized into three groups, namely, low, moderate, and high. Low PA was considered the reference group. For the entire population, subjects who had high PA levels showed significantly reduced risks of GC in model 2 (OR = 0.59, 95% CI = 0.43–0.83) and model 3 (OR = 0.61, 95% CI = 0.43–0.87). The trends were significant in both models (*p*-trend = 0.007 and 0.014, respectively). A similar inverse association between exercise and GC risk was observed for females in model 2 (OR = 0.52, 95% CI = 0.28–0.96, *p*-trend = 0.038) and model 3 (OR = 0.51, 95% CI = 0.26–0.98, *p*-trend = 0.043). However, the reduction in GC risk with exercise was not significant for males. Table S2 shows the corresponding results after stratification by *H. pylori* infection status (positive or negative). In the *H. pylori*-positive group, a significant inverse association between high PA level and the risk of GC was observed in model 2 (OR = 0.61, 95% CI = 0.42–0.89, *p*-trend = 0.016). However, when considering males and females separately, the reductions in GC risk with PA were not significant. In the *H. pylori*-negative group, none of the associations were significant, although reduced risks were observed.

Table 4 presents the associations between each of PA (based on IPAQ-SF continuous score) and MET minutes per week and GC risk. Subjects who were in the highest tertile of low-intensity activity, which represents walking MET minutes per week, showed a significantly reduced risk of GC in model 2 (OR = 0.45, 95% CI = 0.33–0.62, *p*-trend < 0.001) and model 3 (OR = 0.46, 95% CI = 0.33–0.65, *p*-trend < 0.001). Subjects who were in a high category of moderate-intensity activity showed a significantly reduced risk of GC in model 2 (OR = 0.69, 95% CI = 0.52–0.93, *p*-trend = 0.013) and model 3 (OR = 0.69, 95% CI = 0.51–0.94, *p*-trend = 0.019). Similarly, subjects who were in a high category of vigorous-intensity activity (VA) showed a significant inverse association in model 2 (OR = 0.58, 95% CI = 0.43–0.78, *p*-trend < 0.001) and model 3 (OR = 0.61, 95% CI = 0.48–0.84, *p*-trend = 0.002). Similarly, subjects who had high levels of total PA showed a significantly reduced risk of GC relative to those in the lowest tertile in model 2 (OR = 0.47, 95% CI = 0.34–0.64, *p*-trend < 0.001) and model 3 (OR = 0.46, 95% CI = 0.32–0.65, *p*-trend < 0.001).

In males, those who were in the highest tertile of low-intensity PA showed a significantly reduced risk of GC in model 2 (OR = 0.57, 95% CI = 0.39–0.85, *p*-trend = 0.032) and model 3 (OR = 0.60, 95% CI = 0.39–0.91, *p*-trend = 0.078). Those who were in a high category of moderate-intensity activity showed a significantly reduced risk of GC in model 2 (OR = 0.59, 95% CI = 0.41–0.86, *p*-trend = 0.006) and model 3 (OR = 0.57, 95% CI = 0.38–0.86, *p*-trend = 0.006). Accordingly, males who were in the highest tertile of total PA showed an inverse association with GC risk in model 2 (OR = 0.62, 95% CI = 0.41–0.93, *p*-trend = 0.036) and model 3 (OR = 0.62, 95% CI = 0.40–0.96, *p*-trend = 0.054).

In females, those who were in the highest tertile of low-intensity activity showed a significantly reduced GC risk in model 2 (OR = 0.44, 95% CI = 0.25–0.76, *p*-trend = 0.004) and model 3 (OR = 0.44, 95% CI = 0.25–0.81, *p*-trend = 0.014). In contrast, those who engaged in a high level of moderate-intensity activity did not show a significantly reduced GC risk, although a reduced risk was observed. However, those who were in the high category of VA showed a significant risk reduction in model 2 (OR = 0.41, 95% CI = 0.22–0.75, *p*-trend = 0.004) and model 3 (OR = 0.48, 95% CI = 0.25–0.91, *p*-trend = 0.026). Similarly, those who were in the highest tertile of total PA showed a significantly reduced GC risk in model 2 (OR = 0.40, 95% CI = 0.23–0.70, *p*-trend = 0.001) and model 3 (OR = 0.43, 95% CI = 0.23–0.79, *p*-trend = 0.005).

Table S3 presents the corresponding results by *H. pylori* status. In the *H. pylori*-positive group, those who were in the highest tertile of low-intensity activity showed a significantly reduced risk of GC in model 2 (OR = 0.47, 95% CI = 0.33–0.69, *p*-trend < 0.001). Additionally, in the highest tertile of low-intensity type of activity, significant associations were observed for males (OR = 0.59, 95% CI = 0.38–0.93) and females (OR = 0.50, 95% CI = 0.26–0.95), although the reduction trends (*p*-trends) were not significant: 0.088 and 0.072 for males and females respectively. Regarding moderate-intensity activity, those who were in a high category showed a significant reduction in risk (OR = 0.67, 95% CI = 0.48–0.93, *p*-trend = 0.015). The corresponding reduced risk was significant for males (OR = 0.55, 95% CI = 0.36–0.84, *p*-trend = 0.006) but not females. Those who were in the highest category of VA showed a significantly reduced GC risk (OR = 0.61, 95% CI = 0.43–0.85, *p*-trend = 0.004). The reduction in risk was significant for females (OR = 0.48, 95% CI = 0.24–0.98, *p*-trend = 0.044) but not males. With regard to total PA, subjects who were in the highest tertile of total PA showed a significantly reduced risk of GC (OR = 0.44, 95% CI = 0.31–0.64, *p*-trend < 0.001). The association was significant in males (OR = 0.61, 95% CI = 0.38–0.97), although the reduction trend showed marginal significance (*p*-trend = 0.064), whereas females showed a significantly reduced risk (OR = 0.43, 95% CI = 0.22–0.83, *p*-trend = 0.009). In the *H. pylori*-negative group, those who were in the highest tertile of the low-intensity activity group showed a significantly reduced risk of GC (OR = 0.30, 95% CI = 0.10–0.84, *p*-trend = 0.032). However, significant associations were not observed for the moderate, vigorous, and total PA components. Furthermore, regardless of PA type, none of the associations were significant in males. In contrast, in females, those who were in the highest tertile of low-intensity activity showed a marginally significant reduced risk in model 2 (OR = 0.14, 95% CI = 0.02–1.09, *p*-trend = 0.049).

## 3. Discussion

In this case-control study involving 1245 participants (415 cases and 830 controls), we observed a significant inverse association between PA and the risk of GC. PA was assessed in terms of a categorical score and a continuous score (MET minutes per week) on the IPAQ-SF. We also observed an association for each sex. In addition, we performed a subgroup analysis based on *H. pylori* infection status, i.e., positive or negative status, as *H. pylori* infection is a strong risk factor for GC. There was a significant inverse association between regular exercise and GC risk for the entire study population and for males and females separately in the multivariate adjusted model 3. According to the IPAQ-SF categorical score, a high level of PA was associated with a significant reduction in GC risk for the entire population, and for females alone in the multivariate-adjusted model 3. Based on the IPAQ-SF continuous score (MET minutes per week), each type of activity, namely, low-intensity (representing walking), moderate-intensity, high-intensity, and total PA (representing the summation of the other three types of activities), showed a significant inverse association with GC risk for the entire study population.

Engaging in PA has been shown in previous epidemiological studies to reduce the risk of GC [32,33,34], consistent with the results of the current study. According to previous meta-analyses and systematic reviews, significant proportions of people with extremely active lifestyles show reduced risks of GC [35,36,37,38]. Although the latest analysis of global research in the report on stomach cancer [39] published by the World Cancer Research Fund International Continuous Update Project indicated that there is limited evidence to conclude that PA has a protective effect against GC, the promotion of PA is a key indicator of the prevention of several chronic diseases, such as cancer, cardiovascular diseases, and diabetes. Physical activity is also a significant contributor to well-being and healthy aging [40]. We observed that when people engage in regular exercise, the risk of GC can be reduced by approximately 50%. The reduced risks were significant for both males and females. A prospective study of the European Prospective Investigation into Cancer and Nutrition (EPIC) cohort revealed a lower risk of overall and non-cardia GC among individuals with increasing levels of a PA index that combined occupational PA with weekly time spent in sports and cycling (hazard ratio (HR) = 0.69, 95% CI: 0.50–0.94). A comparison between active and inactive participants according to PA index yielded the following results: HR = 0.44 and 95% CI: 0.26–0.74 for non-cardia GC [41]. In the analyses considering *H. pylori* infection status, a significant inverse association between PA and GC risk was observed in the *H. pylori*-positive group, whereas the association, although inverse, was nonsignificant in the *H. pylori*-negative group. This nonsignificant association in the *H. pylori*-negative group could be attributed to reduced statistical power, as there was a smaller number of case subjects in the *H. pylori*-negative subgroup than in the *H. pylori*-positive subgroup.

Regarding IPAQ-SF categorical score in the current study, subjects in the entire study population who had high PA levels in terms of frequency and duration showed inverse associations with the risk of GC. Accordingly, females who had high levels of PA showed a significantly reduced risk of GC. Interestingly, a null association between high PA level and GC risk was observed for the male group in the multivariate-adjusted model 3. A similar result was observed in a large-scale population-based cohort study in Japan [42], in which a decreased risk of stomach cancer with PA was more clearly observed in women than in men, especially among the elderly and those who regularly engaged in leisure-time sports or physical exercise. A possible biological mechanism underlying these observations is the alteration of sex hormones due to PA behaviors [43]. After stratifying the population by *H. pylori* infection status, a significantly reduced risk of GC was observed only among subjects with high PA levels in the *H. pylori*-positive group. However, the reduction in risk was nonsignificant when considering males and females separately. Additionally, none of the associations were significant in the *H. pylori*-negative infection group.

The measurement of PA was based on IPAQ-SF continuous score, calculated in MET minutes per week. Subjects in the highest tertile (≥2970 MET minutes per week) of total PA showed a significantly reduced risk of GC. Significant reductions in risk were observed in individuals with high levels of other specific types of activities, namely, low-intensity, moderate-intensity, and vigorous-intensity activities. According to a case-control study conducted in Spain [44], in which PA was estimated in MET minutes per week, the highest household PA category showed a strong inverse association with GC (OR = 0.50, 95% CI = 0.38–0.66). Recreational PA was also associated with lower overall GC risk (OR = 0.68, 95% CI = 0.52–0.88), particularly at moderate levels of intensity, such as walking (OR = 0.61, 95% CI = 0.46–0.79) [44]. Following stratification by sex, both male and female subjects who were in the highest tertile of the total PA category showed significant inverse associations with GC risk. Sex-specific cutoff values of MET minutes per week were used in the analyses by sex. Therefore, the male group had a higher value for the highest tertile of total PA (≥3390 MET minutes per week) than did the female group (≥2128 MET minutes per week). This finding indicates that the men were more active than the women in the study population. A study based on the association between PA (according to type and intensity) and digestive system cancer risk concluded that aerobic exercise was particularly beneficial against digestive system cancers, with the optimal benefit observed at approximately 30 MET hours per week (HR = 0.68, 95% CI = 0.56–0.83) [45]. Another study revealed that moderate-to-vigorous PA was associated with a 20% to 30% reduction in the risk of gastroesophageal adenocarcinomas, with a significant dose response relationship, and the benefit was greater in women than in men [46].

Following stratification by *H. pylori* infection status, significant inverse associations were observed in subjects in the highest tertile of total PA in the *H. pylori*-positive group, whereas null associations were observed in the *H. pylori*-negative group. Similar associations were observed for different levels of regular exercise and for IPAQ-SF categorical scores. These findings could be explained by the limited sample size in the *H. pylori*-negative group, which led to low statistical power. Additionally, PA did not protect against *H. pylori* infection or gastric ulceration; however, the biological mechanisms related to the impacts of exercise on immune function, antioxidant activity, and gastroesophageal reflux need to be explored [46]. Regarding the biological roles of innate immunity in infection and cancer, *H. pylori* produces an inflammatory response in the gastric mucosa. Following *H. pylori* infection, innate and acquired immune responses are induced in the stomach and are characterized by activation of macrophages and dendritic cells, the production of antibodies, and the differentiation and activation of different effector T cells. For instance, Interferon-γ (IFN-γ) secreting T helper 1 (Th1) cells play a vital role in the response to *H. pylori* infection [47].

The peptidyl prolyl cis, trans-isomerase (PPIase) HP0175 is one of the bacterial antigens secreted by *H. pylori* that can be recognized by sera of *H. pylori*-infected patients. There is extensive coordination of T cells in *H. pylori* infection. In vivo studies of the stomach of humans and animal models demonstrated the production of IFN-γ, Interleukin (IL)-12, IL-18, IL-23, and Tumor Necrosis Factor-α (TNF-α) due to the activation of Th cells. This type of immune response is expected to play a role in the pathogenesis of *H. pylori*-associated disease in humans, particularly inflammation leading to GC [47]. Tissue macrophages are largely inaccessible in humans; however, a study of animal models (utilizing peritoneal macrophages) revealed that resident macrophages have low functional activity in the peritoneum [48]. However, inflammatory agents or antigens can elicit the movement of macrophages to the peritoneum increasing cell yield and their responsiveness to priming signals (e.g., IFN-γ). IFN-γ, an inflammatory cytokine produced by activated T cells, primes macrophages for antitumor and microbial activity by increasing their sensitivity to lipopolysaccharides due to the influence of inflammatory agents [48]. These biological changes produce a pro-inflammatory response that links *H. pylori* bacterial infection with GC occurrence.

Interestingly, a study focused on exercise in regulation of inflammatory-immune axis function in cancer initiation and progression noted that exercise induces the activation of the hypothalamic-pituitary-adrenal axis, which facilitates the release of cortisol and catecholamines [49]. These chemicals can downregulate the lipopolysaccharide-induced production of inflammatory cytokines by antigen-presenting cells such as macrophages and dendritic cells. Such alterations cumulatively result in an anti-inflammatory systemic host environment that can be maintained due to long-term, sustained exercise [49]. Thus, exercise can have a protective effect for the risk of GC by creating an anti-inflammatory response to control the pro-inflammatory response induced by bacterial infection, particularly *H. pylori* infection. Furthermore, several studies have observed exercise in several different species (humans, mice) to enhance a variety of macrophage capacities, including chemotaxis, phagocytic and cytotoxic activity [50,51,52]. One study identified the mechanism responsible for the exercise-induced increase in phagocytosis [53].

Identifying the biological mechanism underlying the link between PA and GC is necessary; however, evidence is lacking. One possible mechanism is the mediation of a pro-inflammatory pathway by the cyclooxygenase-2 (COX-2) enzyme. A functional polymorphism in the COX-2 gene has been associated with an increased risk of digestive cancer, including GC [54]. Thus, factors that affect the COX-2 pathway can modify the risk of GC. PA has been identified as one of the potential candidates that can reduce the activity of the COX-2 pro-inflammatory pathway to decrease the risk of GC [45]. Furthermore, PA can reduce systemic inflammation, which can reduce overall cancer risk [55]. Other mechanisms and enhancing effects on the immune system, including increases in the levels of circulating tumor-inhibiting natural killer cells and their cancer-inhibiting abilities, have been proposed [56]. Additionally, physical exertion upregulates the activity of free scavenger systems and oxidant levels to maintain oxidative stress in the body. Decreases in insulin and insulin-like growth factors are also plausible ways in which protective effects could be mediated [57].

The current study has several strengths. We collected information from the study participants about the prevalence of *H. pylori* infection, which has been identified as a strong risk factor for GC. Additionally, we adjusted for possible confounding variables in models of the association, particularly *H. pylori* infection. In the current study, we also performed analyses by *H. pylori* infection status. However, this study has some potential limitations. Given that this is a hospital-based case-control study, the potential presence of bias, including selection bias and recall bias, must be acknowledged. In addition, subject recall of regular exercise and PA habits may have differed between the case subjects and controls or between males and females due to different levels of health and behavior compliance. Furthermore, the case subjects were more prone to recall this information as they tended to emphasize their lack of physical activity. Moreover, the lack of experimental data based on in vivo models is a limitation, although *H. pylori* infection was assessed experimentally using a rapid urease test and histological evaluation in the current study.

## 4. Materials and Methods

### 4.1. Study Population

**Figure 1:**
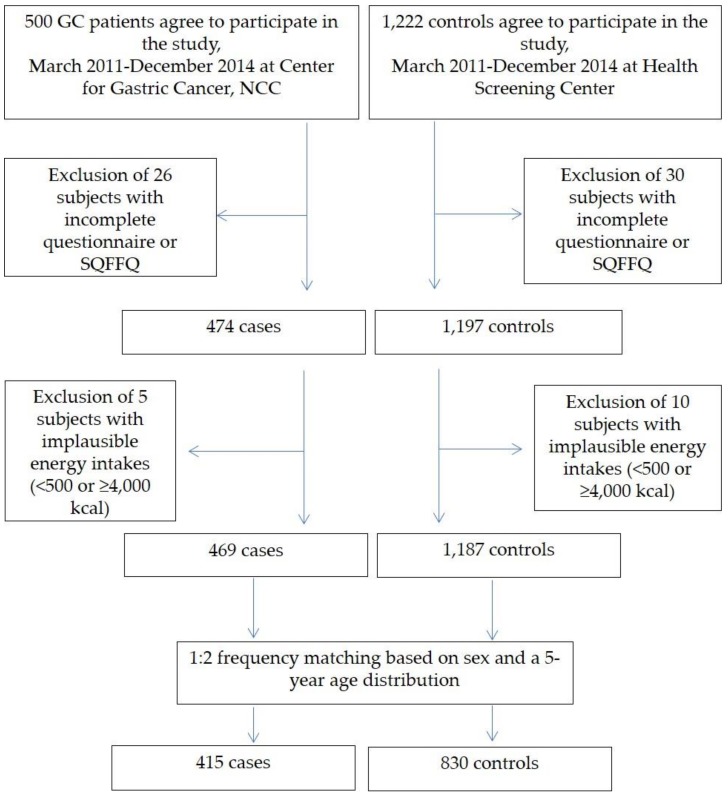
Simplified flow chart for the selection of study subjects.

This study is an extension of three previously published case-control studies [58,59,60]. Participants were recruited at the National Cancer Center Hospital in Korea between March 2011 and December 2014. Individuals who had been histologically confirmed as early GC patients within the preceding three months at the Center for Gastric Cancer were included in the case group. Early GC was defined as an invasive carcinoma confined to the mucosa and/or submucosa regardless of lymph node metastasis status [61]. Patients diagnosed with diabetes mellitus, a history of cancer within the past five years, advanced GC, or severe systemic or mental disease were excluded, as were women who were pregnant or breastfeeding. The control group was selected from patients subjected to health-screening examinations at the Center for Cancer Prevention and Detection at the same hospital. Individuals with a history of cancer, diabetes mellitus, gastric ulcers, and *H. pylori* treatment were excluded from the control group. In total, 1727 participants were recruited (1227 controls and 500 GC patients), and 1671 individuals provided data through a semi-quantitative food frequency questionnaire (SQFFQ) and a self-administered questionnaire. Individuals with a total energy intake of <500 kcal or ≥4000 kcal (*n* = 15) were excluded because of the reliability of the data. Of the 1656 participants remaining, the control and case groups were frequency-matched by age (within 5 years) and sex at a ratio of 2:1 (controls/GC patients). The final sample included 1245 participants composed of 830 controls and 415 GC patients (men, 810; women, 435) [60]. Figure 1 shows a simplified flowchart describing the selection of study subjects). This study was approved by the Institutional Review Board of the National Cancer Center (IRB Number: NCCNCS-11-438, Permission date (7 March 2011)). Written informed consent was obtained from all participants.

### 4.2. Data Collection

Participants were asked to complete a self-administered questionnaire. Demographic, lifestyle, and medical history data were collected from the participants. Total energy intake was obtained from the SQFFQ, which has been previously reported as a reliable and valid questionnaire [62]. The SQFFQ includes nine food consumption frequency categories (i.e., never or rarely, once a month, two or three times a month, once or twice a week, three or four times a week, five or six times a week, once a day, twice a day, and three times a day) and three portion-size categories (i.e., small, medium, and large) for specific food items consumed within the past 12 months. The average daily nutrient intake for each participant was calculated using CAN-PRO 4.0 (Computer Aided Nutritional Analysis Program, Korean Nutrition Society, Seoul, Korea). We summed the amounts of energy obtained from various food groups to compute total energy intake (kcal/day). *H. pylori* infection was assessed by a rapid urease test and histological evaluation.

### 4.3. Assessment of PA: International Physical Activity Questionnaire–Short Form (IPAQ-SF)

The IPAQ-SF was extensively developed as a surveillance instrument for the standardized measurement of PA behaviors in different populations [63,64,65,66]. The IPAQ-SF was used to measure PA over the past seven days and included a seven-item short self-report form [63]. The IPAQ-SF consists of items assessing the frequency and duration of PA during the last seven days in three categories of intensity: VA = 8.0 METs), moderate-intensity activity (MA = 4.0 METs), and low-intensity activity (LA = 3.3 METs), undertaken during a typical week across a set of domains, including leisure time, domestic tasks and gardening (yard work), and work-related and transport-related PA. The total PA score was calculated using standardized IPAQ-SF scoring protocols (www.ipaq.ki.se) to yield total MET minutes per week of PA. Based on the collected frequency and duration of PA and an estimate of energy expenditure expressed in MET minutes per week, the participants were classified into three groups according to their PA levels: (1) high physical activity level (HPAL), which corresponded to any one of the following criteria: three or more days of VA of at least 1500 MET minutes per week or seven or more days of any combination of activities of three intense categories of at least 3000 MET minutes per week; (2) moderate physical activity level (MPAL), which corresponded to three or more days of VA of at least 20 min/day, five or more days of moderate- and/or low-intensity activity of at least 30 min/day, or five or more days of any combination of low-, moderate-, or VA of at least 600 MET minutes per week; and (3) low physical activity level (LPAL), which corresponded to no activity reported or some activity reported but not enough to meet the MPAL criteria. The reliability and validity of the IPAQ have been tested for adults in 12 countries [63]. Adequate reliability (*r* = 0.76), convergent validity against accelerometer data (*r* = 0.3), and concurrent validity (*r* = 0.67) have been established for the IPAQ-SF for sighted individuals [63].

### 4.4. Statistical Analyses

To compare the demographic and lifestyle characteristics between the control and case groups, the chi-square test and Student’s *t*-test were performed for categorical variables and continuous variables, respectively. The Mann-Whitney U test was applied to compare the continuous scores on the IPAQ (MET minutes per week) between the control and case groups due to the non-normal distribution of the variables. To investigate the association between regular exercise and GC risk, a group of subjects who did not regularly exercise served as the reference group. A group of subjects with low PA levels served as the reference group to investigate the association between PA and GC risk according to the IPAQ-SF categorical score. To investigate the association between PA and GC risk in terms of the IPAQ-SF continuous score, each PA type was categorized into tertiles according to the distribution of MET minutes per week in the control group. The lowest tertile of MET minutes per week was used as the reference group. For both moderate- and vigorous-intensity activities, the first tertile (33.33rd percentile) value was zero (0). Thus, the activities with these intensities were divided into two categories: low and high. Accordingly, the low group was considered to be the reference group. The odds ratios (ORs) and 95% confidence intervals (CIs) were estimated using unconditional logistic regression models. The median values of MET minutes per week in each category were treated as continuous variables to test for trends. The OR estimates were calculated for three models, namely, crude, model 2, and model 3. Model 2 was adjusted for smoking, first-degree family history of GC, education, occupation, monthly income, and total energy intake, whereas model 3 was additionally adjusted for *H. pylori* infection status. Subgroup analysis was performed after stratification by sex and *H. pylori* infection (positive or negative).

## 5. Conclusions

In conclusion, our study identified an inverse association between PA and GC risk in the entire study population. Furthermore, an inverse association was observed within the *H. pylori*-positive group. Our findings indicate a need for the promotion of all domains of PA, especially for general Korean populations. Thus, we recommend that epidemiological studies, such as cohort studies, be conducted to verify these results and to determine the biological mechanisms associated with the protective effects of PA.

## Figures and Tables

**Table 1:** General characteristics of the study population.

Variable	All (*n* = 1245)			Male (*n* = 810)			Female (*n* = 435)		
	Control group (*n* = 830)	Case group (*n* = 415)	*p*-value ^b^	Control group (*n* = 540)	Case group (*n* = 270)	*p*-value ^b^	Control group (*n* = 290)	Casegroup (*n* = 145)	*p*-value ^b^
Age (y)	53.7 ± 9.0	53.8 ± 9.3	0.892	54.8 ± 8.4	54.9 ± 8.7	0.905	51.6 ± 9.8	51.7 ± 9.9	0.942
<50	285 (34.3)	139 (33.5)	0.767	153 (28.33)	77 (28.5)	0.956	132 (45.5)	62 (42.8)	0.585
≥50	545 (65.7)	276 (66.5)		387 (71.7)	193 (71.5)		158 (54.5)	83 (57.2)	
Gender [*n* (%)]			0.9999						
Male	540 (65.1)	270 (65.1)							
Female	290 (34.9)	145 (34.9)							
Body mass index (kg/m²) [n (%)]	23.9 ± 2.9	23.9 ± 3.0	0.627	24.4 ± 2.7	24.2 ± 3.0	0.390	23.1 ± 3.1	23.2 ± 3.0	0.788
<23	314 (37.8)	159 (38.3)	0.975	161 (29.8)	91 (33.7)	0.509	153 (52.8)	68 (46.9)	0.533
23–25	249 (30.0)	122 (29.4)		170 (31.5)	78 (28.9)		79 (27.2)	44 (30.3)	
≥25	266 (32.1)	133 (32.1)		209 (38.7)	101 (37.4)		57 (19.7)	32 (22.1)	
Missing	1 (0.1)	1 (0.2)		0 (0.0)	0 (0.0)		1 (0.3)	1 (0.7)	
Smoking status [*n* (%)]			<0.001			<0.001			0.021
Current smoker	162 (19.5)	128 (30.8)		157 (29.1)	121 (44.8)		5 (1.7)	7 (4.8)	
Ex-smoker	284 (34.2)	119 (28.7)		277 (51.3)	110 (40.7)		7 (2.4)	9 (6.2)	
Non-smoker	384 (46.3)	167 (40.2)		106 (19.6)	39 (14.4)		278 (95.9)	128 (88.3)	
Missing	0 (0.0)	1 (0.2)		0 (0.0)	0 (0.0)		0 (0.0)	1 (0.7)	
Alcohol consumption [*n* (%)]			0.243			0.282			0.819
Current drinker	534 (64.3)	254 (61.2)		404 (74.8)	193 (71.5)		130 (44.8)	61 (42.1)	
Ex-drinker	60 (7.2)	41 (9.9)		47 (8.7)	33 (12.2)		13 (4.5)	8 (5.5)	
Non-drinker	236 (28.4)	119 (28.7)		89 (16.5)	44 (16.3)		147 (50.7)	75 (51.7)	
Missing	0 (0.0)	1 (0.2)		0 (0.0)	0 (0.0)		0 (0.0)	1 (0.7)	
First-degree family history of gastric cancer			<0.001			0.003			0.114
Yes	103 (12.4)	82 (19.8)		74 (13.7)	60 (22.2)		29 (10.0)	22 (15.2)	
No	725 (87.4)	332 (80.0)		464 (85.9)	209 (77.4)		261 (90.0)	123 (84.8)	
Missing	2 (0.2)	1 (0.2)		2 (0.4)	1 (0.4)		0 (0.0)	0 (0.0)	
Regular exercise [*n* (%)]			<0.001			<0.001			<0.001
Yes	466 (56.1)	147 (35.4)		303 (56.1)	109 (40.4)		163 (56.2)	38 (26.2)	
No	361 (43.4)	268 (64.6)		234 (43.3)	161 (59.6)		127 (43.8)	107 (73.8)	
Missing	3 (0.4)	0 (0.0)		3 (0.6)	0 (0.0)		0 (0.0)	0 (0.0)	
Educational level [*n* (%)]			<0.001			<0.001			<0.001
Middle school	119 (14.3)	142 (34.2)		71 (13.2)	91 (33.7)		48 (16.6)	51 (35.2)	
High school	253 (30.5)	174 (41.9)		140 (25.9)	112 (41.5)		113 (38.9)	62 (42.8)	
College or more	426 (51.3)	97 (23.4)		301 (55.7)	66 (24.4)		125 (43.1)	31 (21.4)	
Missing	32 (3.9)	2 (0.5)		28 (5.2)	1 (0.4)		4 (1.4)	1 (0.7)	
Occupation [*n* (%)]			<0.001			0.009			0.002
Group 1: Professionals, administrative management	156 (18.8)	70 (16.9)		117 (21.7)	59 (21.9)		39 (13.5)	11 (7.6)	
Group 2: Office, sales, and service positions	266 (32.1)	122 (29.4)		203 (37.6)	81 (30.0)		63 (21.7)	41 (28.3)	
Group 3:Agriculture, laborer	128 (15.4)	104 (25.1)		111 (20.6)	83 (30.7)		17 (5.9)	21 (14.5)	
Group 4:Unemployment and others	277 (33.4)	117 (28.2)		106 (19.6)	46 (17.0)		171 (58.9)	71 (49.0)	
Missing	3 (0.4)	2 (0.5)		3 (0.6)	1 (0.4)		0 (0.0)	1 (0.7)	
Marital status [*n* (%)]			0.611			0.475			0.975
Married	716 (86.3)	361 (87.0)		478 (88.5)	243 (90.0)		238 (82.1)	118 (81.4)	
Others (single, divorced, separated, widowed, cohabitating)	113 (13.6)	52 (12.5)		61 (11.3)	26 (9.6)		52 (17.9)	26 (17.9)	
Missing	1 (0.1)	2 (0.5)		1 (0.2)	1 (0.4)		0 (0.0)	1 (0.7)	
Monthly income [*n* (%)] ^a^			<0.001			<0.001			0.016
<200	149 (18.0)	133 (32.1)		85 (15.7)	85 (31.5)		64 (22.1)	48 (33.1)	
200–400	341 (41.1)	148 (35.7)		232 (43.0)	106 (39.3)		109 (37.6)	42 (28.9)	
≥400	273 (32.9)	96 (23.1)		168 (31.1)	55 (20.4)		105 (36.2)	41 (28.3)	
Missing	67 (8.1)	38 (9.2)		55 (10.2)	24 (8.9)		12 (4.1)	14 (9.7)	
*H. pylori* infection			<0.001			<0.001			<0.001
Positive	486 (58.6)	382 (92.1)		333 (61.7)	252 (93.3)		153 (52.8)	130 (89.7)	
Negative	320 (38.6)	33 (8.0)		187 (34.6)	18 (6.7)		133 (45.9)	15 (10.3)	
Missing	24 (2.9)	0 (0.0)		20 (3.7)	0 (0.0)		4 (1.4)	0 (0.0)	
Supplements use [*n* (%)]			0.094			0.193			0.291
Yes	527 (63.5)	243 (58.6)		329 (60.9)	152 (56.3)		198 (68.3)	91 (62.8)	
No	298 (35.9)	169 (40.7)		206 (38.2)	116 (43.0)		92 (31.7)	53 (36.6)	
Missing	5 (0.6)	3 (0.7)		5 (0.9)	2 (0.7)		0 (0.0)	1 (0.7)	
Lauren’s classification									
Intestinal	NA	158 (38.1)	NA	NA	132 (48.9)	NA	NA	26 (17.9)	NA
Diffuse	NA	164 (39.5)		NA	77 (28.5)		NA	87 (60.0)	
Mixed	NA	59 (14.2)		NA	40 (14.8)		NA	19 (13.1)	
Intermediate	NA	4 (1.0)		NA	3 (1.1)		NA	1 (0.7)	
Missing	NA	30 (7.2)		NA	18 (6.7)		NA	12 (8.3)	
IPAQ categorical score			0.005			0.026			0.152
Low	273 (32.9)	175 (42.2)		163 (30.2)	106 (39.3)		110 (37.9)	69 (47.6)	
Moderate	309 (37.2)	128 (30.8)		191 (35.4)	77 (28.5)		118 (40.7)	51 (35.2)	
High	248 (29.9)	112 (27.0)		186 (34.4)	87 (32.2)		62 (21.4)	25 (17.2)	
IPAQ Continuous Score ^c^									
Walking MET min/week	1203.0 ± 1133.6	973.3 ± 1129.8	0.001	1260.2 ± 1153.3	1042.1 ± 1182.7	0.015	1096.1 ± 1089.9	845.8 ± 1016.4	0.026
Moderate MET min/week	599.7 ± 1021.5	608.6 ± 1174.8	0.897	700.6 ± 1103.7	668.0 ± 1242.9	0.720	411.5 ± 816.2	498.4 ± 1032.4	0.385
Vigorous MET min/week	951.9 ± 1775.8	962.4 ± 2047.3	0.929	1169.0 ± 1970.2	1276.9 ± 2342.7	0.518	540.2 ± 1234.0	377.2 ± 1126.5	0.186
Total MET min/week	2671.1 ± 2829.0	2460.8 ± 3225.8	0.260	3040.9 ± 3066.0	2896.5 ± 3556.1	0.570	1979.3 ± 2162.4	1652.6 ± 2301.6	0.148
Total energy intake (Kcal/day)	1713.6 ± 545.5	1924.1 ± 612.9	<0.001	1760.6 ± 541.5	2038.5 ± 634.8	<0.001	1626.0 ± 543.1	1711.1 ± 507.0	0.116

Values are expressed in mean ± standard deviation (SD) or n (%). IPAQ = International Physical Activity Questionnaire, MET = Metabolic Equivalents. ^a^ Unit is 10,000 Won in Korean currency. ^b^ *p*-values refer to the difference between the case and control groups. Age, body mass index (continuous), and total energy intake were examined using Student’s *t*-tests; other variables were assessed using chi-square analysis. ^c^ IPAQ [MET-min/week] was assessed using the Mann-Whitney U test. IPAQ categorical score: Three levels of physical activity were considered: (1) Low: No activity reported, or some activity reported but not enough to meet category 2 or 3. (2) Moderate: Any one of the following three criteria: (a) three or more days of vigorous intensity activity of at least 20 min per day, (b) five or more days of moderate intensity activity and/or walking of at least 30 min per day, or (c) five or more days of any combination of walking, moderate-intensity, or vigorous-intensity activities achieving at least 600 MET min/week. High: Any one of the following two criteria: (a) vigorous intensity activity on at least three days accumulating at least 1500 MET min/week, or (b) seven or more days of any combination of walking, or moderate- or vigorous-intensity activities accumulating at least 3000 MET min/week.

**Table 2:** Association between regular exercise and gastric cancer risk.

Regular Exercise	Gastric Cancer Risk				
	Control group (%)	Cases group (%)	Model 1 (95% CI)	Model 2 OR (95% CI)	Model 3 OR (95% CI)
All					
No	361 (43.7)	268 (64.6)	1.00	1.00	1.00
Yes	466 (56.4)	147 (35.4)	0.43 (0.33–0.54)	0.47 (0.36–0.62)	0.52 (0.39–0.69)
Males					
No	234 (43.6)	161 (59.6)	1.00	1.00	1.00
Yes	303 (56.4)	109 (40.4)	0.52 (0.39–0.70)	0.65 (0.46–0.91)	0.73 (0.50–1.05)
Females					
No	127 (43.8)	107 (73.8)	1.00	1.00	1.00
Yes	163 (56.2)	38 (26.2)	0.28 (0.18–0.43)	0.30 (0.18–0.48)	0.34 (0.21–0.57)

Model 1. Crude. Model 2: Adjusted for smoking, first-degree family history of gastric cancer, education, occupation, monthly income, and total energy intake. Model 3: Additionally adjusted for *H. pylori* infection.

**Table 3:** Association between physical activity (IPAQ categorical score) and gastric cancer risk.

IPAQ Categorical Score	Gastric Cancer				
	Control Group (%)	Case Group (%)	Model 1 (95% CI)	Model 2 OR (95% CI)	Model 3 OR (95% CI)
All					
Low	273 (32.9)	175 (42.2)	1.00	1.00	1.00
Moderate	309 (37.2)	128 (30.8)	0.65 (0.49–0.86)	0.64 (0.47–0.87)	0.67 (0.48–0.94)
High	248 (29.9)	112 (27.0)	0.71 (0.53–0.94)	0.59 (0.43–0.83)	0.61 (0.43–0.87)
*p*-trend			0.067	0.007	0.014
Males					
Low	163 (30.2)	106 (39.3)	1.00	1.00	1.00
Moderate	191 (35.4)	77 (28.5)	0.62 (0.43–0.88)	0.62 (0.41–0.94)	0.63 (0.41–0.98)
High	186 (34.4)	87 (32.2)	0.72 (0.51–1.02)	0.67 (0.44–1.00)	0.70 (0.45–1.09)
*p*-trend			0.213	0.141	0.262
Females					
Low	110 (37.9)	69 (47.6)	1.00	1.00	1.00
Moderate	118 (40.7)	51 (35.2)	0.69 (0.44–1.08)	0.66 (0.41–1.07)	0.78 (0.46–1.31)
High	62 (21.4)	25 (17.2)	0.64 (0.37–1.12)	0.52 (0.28–0.96)	0.51 (0.26–0.98)
*p*-trend			0.119	0.038	0.043

Model 1: Crude. Model 2: Adjusted for smoking, first-degree family history of gastric cancer, education, occupation, monthly income, and total energy intake. Model 3: Additionally adjusted for *H. pylori* infection. IPAQ categorical score: Three levels of physical activity were considered: (1) Low: No activity reported, or some activity reported but not enough to meet category 2 or 3. (2) Moderate: Any one of the following three criteria: (a) three or more days of vigorous intensity activity of at least 20 min per day, (b) five or more days of moderate-intensity activity and/or walking of at least 30 min per day, or (c) five or more days of any combination of walking, moderate-intensity or vigorous-intensity activities achieving at least 600 MET min/week. (3) High: Any one of the following two criteria: (a) vigorous intensity activity on at least three days accumulating at least 1500 MET min/week, or (b) seven or more days of any combination of walking, moderate- or vigorous-intensity activities accumulating at least 3000 MET min/week.

**Table 4:** Association between physical activity (IPAQ continuous score, MET minutes per week) and gastric cancer risk.

All	Median Range MET min/Week	Gastric Cancer Risk				
		Control Group (%)	Case Group (%)	Model 1 (95% CI)	Model 2 OR (95% CI)	Model 3 OR (95% CI)
Low-intensity activity						
T1 (0–495)	198.0	237 (29.9)	182 (46.9)	1.00	1.00	1.00
T2 (495–1386)	792.0	274 (34.6)	92 (23.7)	0.44 (0.32–0.59)	0.46 (0.33–0.64)	0.43 (0.30–0.62)
T3 (≥1386)	2079.0	281 (35.5)	114 (29.4)	0.53 (0.40–0.71)	0.45 (0.33–0.62)	0.46 (0.33–0.65)
*p*-trend				<0.001	<0.001	<0.001
Moderate-intensity activity						
Low (0–480)	0.0	509 (63.7)	279 (69.4)	1.00	1.00	1.00
High (≥480)	1200.0	290 (36.3)	123 (30.6)	0.77 (0.60–1.00)	0.69 (0.52–0.93)	0.69 (0.51–0.94)
*p*-trend				0.05	0.013	0.019
Vigorous-intensity activity						
Low (0–720)	0.0	536 (66.3)	302 (73.3)	1.00	1.00	1.00
High (≥720)	1920.0	272 (33.7)	110 (26.7)	0.72 (0.55–0.93)	0.58 (0.43–0.78)	0.61 (0.48–0.84)
*p*-trend				0.013	<0.001	0.002
Total physical activity						
T1 (0–990)	339.0	259 (31.4)	190 (45.9)	1.00	1.00	1.00
T2 (990–2970)	1699.5	290 (35.2)	109 (26.3)	0.51 (0.38–0.68)	0.52 (0.38–0.72)	0.56 (0.40–0.78)
T3 (≥2970)	4878.0	275 (33.4)	115 (27.8)	0.57 (0.43–0.76)	0.47 (0.34–0.64)	0.46 (0.32–0.65)
*p*-trend				0.001	<0.001	<0.001
Males						
Low-intensity activity						
T1 (0–594)	231.0	163 (31.6)	124 (49.2)	1.00	1.00	1.00
T2 (594–1386)	792.0	163 (31.6)	43 (17.1)	0.35 (0.23–0.52)	0.39 (0.25–0.62)	0.38 (0.23–0.62)
T3 (≥1386)	2376.0	190 (36.8)	85 (33.7)	0.59 (0.42–0.83)	0.57 (0.39–0.85)	0.60 (0.39–0.91)
*p*-trend				0.042	0.032	0.078
Moderate-intensity activity						
Low (0–720)	0.0	342 (65.8)	189 (72.4)	1.00	1.00	1.00
High (≥720)	1440	178 (34.2)	72 (27.6)	0.73 (0.53–1.01)	0.59 (0.41–0.86)	0.57 (0.38–0.86)
*p*-trend				0.061	0.006	0.006
Vigorous-intensity activity						
Low (0–960)	0.0	334 (63.1)	181 (67.5)	1.00	1.00	1.00
High (≥960)	2400	195 (36.9)	87 (32.5)	0.82 (0.60–1.12)	0.71 (0.50–1.02)	0.73 (0.50–1.07)
*p*-trend				0.220	0.065	0.109
Total physical activity						
T1 (0–1188)	495.0	173 (32.2)	116 (43.1)	1.00	1.00	1.00
T2 (1188–3390)	2079.0	184 (34.3)	71 (26.4)	0.58 (0.40–0.83)	0.65 (0.43–0.97)	0.65 (0.42–1.00)
T3 (≥3390)	5473.5	180 (33.5)	82 (30.5)	0.68 (0.48–0.97)	0.62 (0.41–0.93)	0.62 (0.40–0.96)
*p*-trend				0.077	0.036	0.054
Females						
Low-intensity activity						
T1 (0–462)	198.0	89 (32.3)	62 (45.6)	1.00	1.00	1.00
T2 (462–1188)	660.0	83 (30.1)	39 (28.7)	0.67 (0.41–1.11)	0.69 (0.40–1.19)	0.56 (0.31–1.01)
T3 (≥1188)	1848.0	104 (37.7)	35 (25.7)	0.48 (0.29–0.80)	0.44 (0.25–0.76)	0.44 (0.25–0.81)
*p*-trend				0.006	0.004	0.014
Moderate-intensity activity						
Low (0–240)	0.0	177 (63.4)	100 (70.9)	1.00	1.00	1.00
High (≥240)	720.0	102 (36.6)	41 (29.1)	0.71 (0.46–1.10)	0.66 (0.41–1.05)	0.72 (0.43–1.20)
*p*-trend				0.127	0.084	0.205
Vigorous-intensity activity						
Low (0–484)	0.0	211 (75.6)	125 (86.8)	1.00	1.00	1.00
High (≥484)	1440.0	68 (24.4)	19 (13.2)	0.47 (0.27–0.82)	0.41 (0.22–0.75)	0.48 (0.25–0.91)
*p*-trend				0.008	0.004	0.026
Total physical activity						
T1 (0–693)	234.5	92 (32.1)	64 (44.1)	1.00	1.00	1.00
T2 (693–2128)	1314.0	99 (34.5)	48 (33.1)	0.69 (0.44–1.12)	0.75 (0.45–1.25)	0.87 (0.50–1.51)
T3 (≥2128)	3555.0	96 (33.5)	33 (22.8)	0.50 (0.30–0.82)	0.40 (0.23–0.70)	0.43 (0.23–0.79)
*p*-trend				0.008	0.001	0.005

Model 1: Crude. Model 2: Adjusted for smoking, first-degree family history of gastric cancer, education, occupation, monthly income, and total energy intake. Model 3: Additionally adjusted for *H. pylori* infection.

## Supplementary Materials

The following are available online at http://www.mdpi.com/2072-6694/10/10/369/s1, Table S1: Association between regular exercise and gastric cancer risk by *H. pylori* status. Table S2: Association between physical activity (IPAQ categorical score) and gastric cancer risk by *H. pylori* status. Table S3: Association between physical activity (IPAQ continuous score, MET minutes per week) and gastric cancer risk by *H. pylori* status.
