# Supplementary material for: Physical Activity and Gastric Cancer Risk in Patients with and without Helicobacter pylori Infection in A Korean Population: A Hospital-Based Case-Control Study
Source: Cancers (Basel). 2018 Oct 2;10(10):369. doi: 10.3390/cancers10100369 (PMC6210303; doi:10.3390/cancers10100369)
Supplement: Supplementary file 1 [file cancers-10-00369-s001.pdf]

Supplementary Materials: Physical Activity and Gastric Cancer Risk in Patients with and without *Helicobacter pylori* Infection in A Korean Population: A Hospital-Based Case-Control Study

Madhawa Neranjan Gunathilake, Jeonghee Lee, Aelee Jang, Il Ju Choi, Young-Il Kim and Jeongseon Kim

Table S1. Association between regular exercise and gastric cancer risk stratified by *H. pylori* status.

Regular Exercise	*H. Pylori* Positive				*H. Pylori* Negative			
	**Control (%)**	**Cases (%)**	**Model 1 (95% CI)**	**Model 2 OR (95% CI)**	**Control (%)**	**Cases (%)**	**Model 1 OR (95% CI)**	**Model 2 OR (95% CI)**
All								
No	223 (46.1)	248 (64.9)	1.00	1.00	130 (40.6)	20 (60.6)	1.00	1.00
Yes	261 (53.9)	134 (35.1)	0.46 (0.35–0.61)	0.52 (0.38–0.70)	189 (59.3)	13 (39.4)	0.45 (0.22–0.93)	0.53 (0.23–1.21)
Male								
No	152 (45.9)	152 (60.32)	1.00	1.00	74 (39.8)	9 (50.0)	1.00	1.00
Yes	179 (54.1)	100 (39.7)	0.56 (0.40–0.78)	0.68 (0.46–1.01)	112 (60.2)	9 (50.0)	0.66 (0.25–1.74)	1.07 (0.30–3.74)
Female								
No	71 (46.4)	96 (73.9)	1.00	1.00	56 (42.1)	11 (73.3)	1.00	1.00
Yes	82 (53.6)	34 (26.2)	0.31 (0.19–0.51)	0.36 (0.21–0.64)	77 (57.9)	4 (26.7)	0.26 (0.08–0.87)	0.14 (0.03–0.68)

Model 1: Crude; Model 2: Adjusted for smoking, first degree family history of gastric cancer, education, occupation, monthly income and total energy intake.

Table S2. Association between physical activity (IPAQ categorical score) and gastric cancer risk stratified by *H. pylori* status.

IPAQ-Categorical Score	***H. Pylori* Positive**				***H. Pylori* Negative**			
	**Control (%)**	**Cases (%)**	**Model 1 (95% CI)**	**Model 2 OR (95% CI)**	**Control (%)**	**Cases (%)**	**Model 1 (95% CI)**	**Model 2 OR (95% CI)**
All								
Low	166 (34.2)	160 (41.8)	1.00	1.00	99 (30.9)	15 (45.5)	1.00	1.00
Moderate	172 (35.4)	120 (31.4)	0.72 (0.53–1.00)	0.71 (0.50–1.02)	128 (40.0)	8 (24.2)	0.41 (0.17–1.01)	0.30 (0.11–0.83)
High	148 (30.5)	102 (26.7)	0.72 (0.51–1.00)	0.61 (0.42–0.89)	93 (29.1)	10 (30.3)	0.71 (0.30–1.66)	0.64 (0.23–1.74)
p-trend			0.087	0.016			0.779	0.811
Male								
Low	103 (30.9)	99 (39.3)	1.00	1.00	54 (28.9)	7 (38.9)	1.00	1.00
Moderate	118 (35.4)	74 (29.4)	0.65 (0.44–0.97)	0.66 (0.41–1.06)	66 (35.3)	3 (16.7)	0.35 (0.09–1.42)	0.15 (0.03–0.81)
High	112 (33.6)	79 (31.4)	0.73 (0.49–1.09)	0.68 (0.42–1.09)	67 (35.8)	8 (44.4)	0.92 (0.31–2.70)	0.89 (0.22–3.66)
p-trend			0.286	0.208			0.685	0.433
Female								
Low	63 (41.2)	61 (46.9)	1.00	1.00	45 (33.8)	8 (53.3)	1.00	1.00
Moderate	54 (35.3)	46 (35.3)	0.88 (0.52–1.49)	0.89 (0.50–1.59)	62 (46.6)	5 (33.3)	0.45 (0.14–1.48)	0.40 (0.10–1.55)
High	36 (23.5)	23 (17.7)	0.66 (0.35–1.24)	0.56 (0.28–1.12)	26 (19.6)	2 (13.3)	0.43 (0.09–2.19)	0.26 (0.03–2.03)
p-trend			0.196	0.103			0.339	0.216

Model 1. Crude; Model 2: Adjusted for smoking, first degree family history of gastric cancer, education, occupation, monthly income, total energy intake; IPAQ categorical score-Three levels of physical activity are proposed:

1) Low
   No activity is reported OR some activity is reported but not enough to meet category 2 or 3
2) Moderate
   Either of the following three criteria;
   a) Three or more days of vigorous intensity activity of at least 20 minutes per day OR
   b) Five or more days of moderate intensity activity and/or walking of at least 30 minutes per day OR
   c) Five or more days of any combination of walking, moderate-intensity or vigorous intensity activities achieving a minimum of at least 600MET min/week
3) High
   Any one of the following two criteria;
   a) Vigorous intensity activity on at least three days and accumulating at least 1500 MET min/week OR
   b) Seven or more days of any combination of walking, moderate or vigorous intensity activities accumulating at least 3000MET min/week.

Table S3. Association between physical activity (IPAQ continuous score, MET minutes per week) and gastric cancer risk stratified by *H. pylori* status.

All	*H. Pylori* Positive				*H. Pylori* Negative			
	**Control (%)**	**Cases (%)**	**Model 1 (95% CI)**	**Model 2 OR (95% CI)**	**Control (%)**	**Cases (%)**	**Model 1 (95% CI)**	**Model 2 OR (95% CI)**
Low-intensity activity								
T1 (0–495) [198.0]	134 (29.0)	168 (46.8)	1.00	1.00	95 (30.8)	14 (48.3)	1.00	1.00
T2 (495–1386) [792.0]	168 (36.4)	86 (24.0)	0.41 (0.29–0.58)	0.42 (0.29–0.62)	99 (32.1)	6 (20.7)	0.41 (0.15–1.11)	0.42 (0.14–1.29)
T3 (≥1386) [2079.0]	160 (34.6)	105 (29.3)	0.52 (0.37–0.73)	0.47 (0.33–0.69)	114 (37.0)	9 (31.0)	0.54 (0.22–1.29)	0.30 (0.10–0.84)
p-trend			0.001	<0.001			0.249	0.032
Moderate-intensity activity								
Low (0–480) [0.0]	296 (63.4)	260 (69.9)	1.00	1.00	193 (62.5)	19 (63.3)	1.00	1.00
High (≥480) [1200.0]	171 (36.6)	112 (30.1)	0.75 (0.56–0.99)	0.67 (0.48–0.93)	116 (37.5)	11 (36.7)	0.96 (0.44–2.09)	0.98 (0.40–2.39)
p-trend			0.048	0.015			0.925	0.962
Vigorous-intensity activity								
Low (0–720) [0.0]	313 (66.2)	279 (73.6)	1.00	1.00	208 (66.7)	23 (69.7)	1.00	1.00
High (≥720) [1920.0]	160 (33.8)	100 (26.4)	0.70 (0.52–0.94)	0.61 (0.43–0.85)	104 (33.3)	10 (30.3)	0.87 (0.40–1.90)	0.72 (0.29–1.75)
p-trend			0.019	0.004			0.725	0.463
Total physical activity								
T1 (0–990) [339.0]	151 (31.3)	176 (46.2)	1.00	1.00	102 (32.1)	14 (42.4)	1.00	1.00
T2 (990–2970) [1699.5]	166 (34.4)	100 (26.3)	0.52 (0.37–0.72)	0.57 (0.40–0.82)	115 (36.2)	9 (27.3)	0.57 (0.24–1.37)	0.41 (0.16–1.09)
T3 (≥2970) [4878.0]	166 (34.4)	105 (27.6)	0.54 (0.39–0.75)	0.44 (0.31–0.64)	101 (31.8)	10 (30.3)	0.72 (0.31–1.70)	0.62 (0.23–1.69)
p-trend			0.001	<0.001			0.584	0.526
Male								
Low-intensity activity								
T1 (0–594) [231.0]	100 (31.6)	116 (49.2)	1.00	1.00	56 (31.1)	8 (50.0)	1.00	1.00
T2 (594–1386) [792.0]	102 (32.2)	42 (17.8)	0.36 (0.23–0.56)	0.39 (0.23–0.65)	54 (30.0)	1 (6.3)	0.13 (0.02–1.07)	0.12 (0.01–1.38)
T3 (≥1386) [2376.0]	115 (36.3)	78 (33.1)	0.59 (0.40–0.87)	0.59 (0.38–0.93)	70 (38.9)	7 (43.8)	0.70 (0.24–2.05)	0.68 (0.16–2.83)
p-trend			0.065	0.088			0.906	0.853
Moderate-intensity activity								
Low (0–720) [0.0]	208 (65.2)	178 (73.0)	1.00	1.00	115 (63.5)	11 (64.7)	1.00	1.00
High (≥720) [1440]	111 (34.8)	66 (27.1)	0.69 (0.48–1.00)	0.55 (0.36–0.84)	66 (36.5)	6 (35.3)	0.95 (0.34–2.67)	0.84 (0.24–2.93)
p-trend			0.050	0.006			0.924	0.784
Vigorous-intensity activity								

Low (0–960) [0.0]	207 (63.7)	170 (68.0)	1.00	1.00	115 (62.5)	11 (61.1)	1.00	1.00
High (≥960) [2400]	118 (36.3)	80 (32.0)	0.83 (0.58–1.17)	0.71 (0.47–1.07)	69 (37.5)	7 (38.9)	1.06 (0.39–2.86)	1.19 (0.36–3.93)
p-trend			0.281	0.097			0.908	0.771
Total physical activity								
T1 (0–1188) [495.0]	104 (31.3)	109 (43.4)	1.00	1.00	64 (34.6)	7 (38.9)	1.00	1.00
T2 (1188–3390) [2079.0]	120 (36.1)	66 (26.3)	0.53 (0.35–0.79)	0.63 (0.39–1.00)	55 (29.7)	5 (27.8)	0.83 (0.25–2.77)	0.58 (0.14–2.42)
T3 (≥3390) [5473.5]	108 (32.5)	76 (30.3)	0.67 (0.45–1.00)	0.61 (0.38–0.97)	66 (35.7)	6 (33.3)	0.83 (0.26–2.61)	0.88 (0.23–3.46)
p-trend			0.123	0.064			0.779	0.992
Female								
Low-intensity activity								
T1 (0–462) [198.0]	42 (28.9)	56 (45.5)	1.00	1.00	46 (35.9)	6 (46.2)	1.00	1.00
T2 (462–1188) [660.0]	53 (36.6)	34 (27.6)	0.48 (0.27–0.87)	0.50 (0.26–0.95)	30 (23.4)	5 (38.5)	1.28 (0.36–4.56)	1.50 (0.34–6.64)
T3 (≥1188) [1848.0]	50 (34.5)	33 (26.8)	0.50 (0.27–0.90)	0.50 (0.26–0.95)	52 (40.6)	2 (15.4)	0.30 (0.06–1.53)	0.14 (0.02–1.09)
p-trend			0.048	0.072			0.117	0.049
Moderate-intensity activity								
Low (0–240) [0.0]	99 (66.9)	91 (71.1)	1.00	1.00	76 (59.4)	9 (69.2)	1.00	1.00
High (≥240) [720.0]	49 (33.1)	37 (28.9)	0.82 (0.49–1.37)	0.72 (0.41–1.27)	52 (40.6)	4 (30.8)	0.65 (0.19–2.22)	0.65 (0.15–2.77)
p-trend			0.453	0.257			0.492	0.560
Vigorous-intensity activity								
Low (0–484) [0.0]	114 (77.0)	113 (87.6)	1.00	1.00	94 (73.4)	12 (80.0)	1.00	1.00
High (≥484) [1440.0]	34 (23.0)	16 (12.4)	0.48 (0.25–0.91)	0.48 (0.24–0.98)	34 (26.6)	3 (20.0)	0.69 (0.18–2.60)	0.28 (0.05–1.56)
p-trend			0.024	0.044			0.585	0.147
Total physical activity								
T1 (0–693) [234.5]	50 (33.1)	58 (44.6)	1.00	1.00	41 (30.8)	6 (40.0)	1.00	1.00
T2 (693–2128) [1314.0]	48 (31.8)	43 (33.1)	0.77 (0.44–1.35)	0.93 (0.51–1.71)	51 (38.4)	5 (33.3)	0.67 (0.19–2.35)	0.53 (0.12–2.34)
T3 (≥2128) [3555.0]	53 (35.1)	29 (22.3)	0.47 (0.26–0.85)	0.43 (0.22–0.83)	41 (30.8)	4 (26.7)	0.66 (0.18–2.54)	0.32 (0.06–1.68)
p-trend			0.012	0.009			0.598	0.198

Model 1: Crude; Model 2: Adjusted for smoking, first degree family history of gastric cancer, education, occupation, monthly income and total energy intake; Values in the parentheses are the cut off values whereas values in the brackets are the median range values. Units are MET minutes per week.

© 2018 by the authors. Licensee MDPI, Basel, Switzerland. This article is an open access article distributed under the terms and conditions of the Creative Commons Attribution (CC BY) license (http://creativecommons.org/licenses/by/4.0/).
